# Novel insights into Notch signaling in tumor immunity: potential targets for cancer immunotherapy

**DOI:** 10.3389/fimmu.2024.1352484

**Published:** 2024-02-20

**Authors:** Man Wang, Fei Yu, Yuan Zhang, Peifeng Li

**Affiliations:** Institute for Translational Medicine, The Affiliated Hospital of Qingdao University, College of Medicine, Qingdao University, Qingdao, China

**Keywords:** Notch signaling, cancer development, tumor microenvironment, cancer immunotherapy, Notch-targeted therapeutics, tumor immunity

## Abstract

Notch signaling pathway is a highly conserved system of cell-to-cell communication that participates in various biological processes, such as stem cell maintenance, cell fate decision, cell proliferation and death during homeostasis and development. Dysregulation of Notch signaling has been associated with many aspects of cancer biology, such as maintenance of cancer stem-like cells (CSCs), cancer cell metabolism, angiogenesis and tumor immunity. Particularly, Notch signaling can regulate antitumor or pro-tumor immune cells within the tumor microenvironment (TME). Currently, Notch signaling has drawn significant attention in the therapeutic development of cancer treatment. In this review, we focus on the role of Notch signaling pathway in remodeling tumor immune microenvironment. We describe the impact of Notch signaling on the efficacy of cancer immunotherapies. Furthermore, we summarize the results of relevant preclinical and clinical trials of Notch-targeted therapeutics and discuss the challenges in their clinical application in cancer therapy. An improved understanding of the involvement of Notch signaling in tumor immunity will open the door to new options in cancer immunotherapy treatment.

## 1 Introduction

Notch proteins are type I transmembrane proteins that were originally discovered in *Drosophila melanogaster* (1). Notch receptors are composed of an extracellular domain that combines with ligands expressed on a neighboring cell, a transmembrane region and an intracellular region that conveys the signal (2). Notch signaling pathway is initiated by binding of canonical ligands (Delta-like ligand 1 (Dll1), Dll3, Dll4, Jagged1 (Jag1), Jag2) to Notch receptors (Notch1, Notch2, Notch3 and Notch4) (3, 4). Although the core Notch signaling cascade seems to be relatively simple, it is actually a complicated and adaptable machinery, which can induce widespread cellular responses (5). Notch signaling is an evolutionarily conserved system of cell-to-cell communication between adjacent cells, affecting many biological processes such as cell fate determination, cell proliferation and differentiation (4, 6). Dysregulation of Notch signaling is involved in various human pathologies, especially cancer (7). Notch signaling pathway participates in many aspects of cancer biology, encompassing stem cell maintenance, cancer angiogenesis, invasion and metastasis (8–11). Notch signaling can play both tumor-promoting and tumor-suppressing roles in diverse types of cancer (12). A growing body of evidence manifests that Notch signaling exerts double-edged sword effects on tumor immunity by regulating the abundance and functionality of diverse immune cells including myeloid-derived suppressor cells (MDSCs), tumor-associated macrophages (TAMs), dendritic cells (DCs) and T cells (13). Accordingly, manipulation of Notch signaling may hold promise as a powerful new approach for cancer treatment (14). Considerable effort has been devoted to designing Notch-targeting therapies. The past decade has seen the emergence of various types of agents therapeutically inhibiting Notch pathway including ligand/receptor-targeted antibodies, γ-secretase inhibitor (GSI) and small molecule inhibitor (5). Nevertheless, due to the intricate roles of Notch signaling in cancer pathogenesis, the goal of developing tumor-selective Notch-directed therapies for clinical use is still challenging.

In the present review, we provide an up-to-date overview of current findings of Notch signaling in cancer and its crosstalk with tumor immune microenvironment. Moreover, the effects of Notch signaling on cancer immunotherapy responses are highlighted. In addition, we summarize recent preclinical and clinical trials of Notch-targeted therapies to offer new ideas and directions for more precise cancer immunotherapy. A better understanding of Notch signaling in cancer will accelerate the research and development of safe and efficacious Notch-targeting drugs for cancer intervention.

## 2 Roles of Notch signaling in cancer pathogenesis

**Figure 1:**
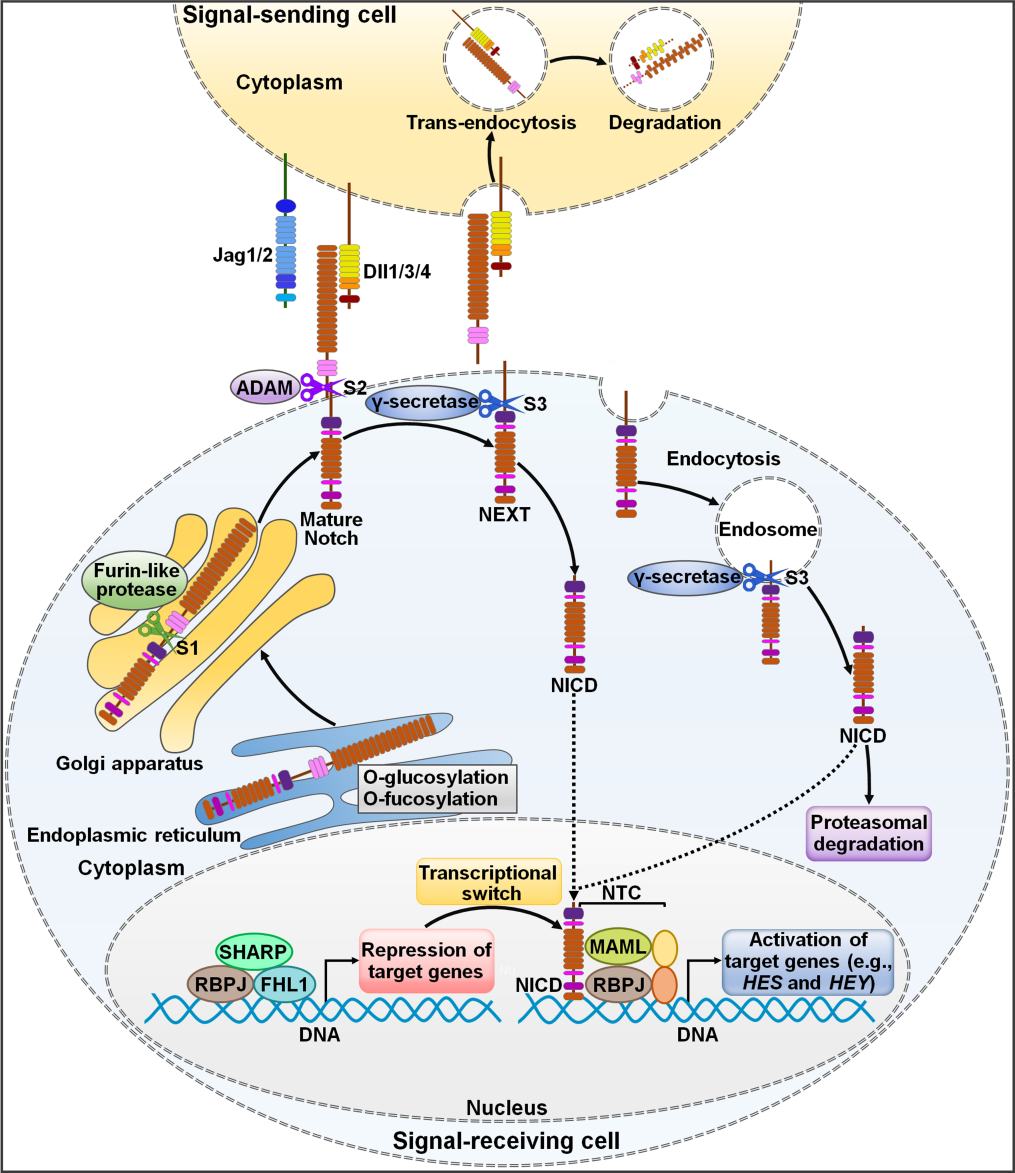
Schematic representation of Notch signaling pathway. The newly translated Notch receptor undergoes protein folding and glycosylation in endoplasmic reticulum. The cleavage of Notch receptor by furin-like protease at S1 occurs in the Golgi apparatus, leading to the biogenesis of mature Notch receptor. It is then transported to the cell membrane as a heterodimer. Notch ligands from signal-sending cells bind to the Notch receptor of signal-receiving cells. Endocytosis and membrane trafficking coordinate the availability of ligands and receptors on the cell surface. Ligand endocytosis may also produce mechanical force to induce structural changes in the bound Notch receptor. This conformational alteration exposes S2 in Notch for sequential cleavages by ADAM, generating a membrane-anchored Notch extracellular truncation fragment (NEXT). γ-Secretase cleaves the Notch transmembrane domain in NEXT at S3, finally releasing the Notch intracellular domain (NICD) from the cell membrane. γ-Secretase-mediated cleavage can occur at the cell surface or in the endosome. NICD translocates to the nucleus and binds to RBPJ, MAML and other proteins to form a NTC that facilitates the transcription of many genes (e.g., *HES* and *HEY*). In the absence of NICD, RBPJ may associate with corepressor proteins to suppress target gene transcription. NICD binding may induce allosteric changes in RBPJ that causes displacement of transcriptional repressors. The transcriptional activator MAML recognizes the NICD/RBPJ interface, following which other coactivators are recruited to activate transcription of downstream target genes. Jag1/2, Jagged1/2; Dll1/3/4, Delta-like ligand 1/3/4; ADAM, a disintegrin and metalloproteinase; S1, Site 1; S2, Site 2; S3, Site 3; NEXT, Notch extracellular truncation; NICD, Notch intracellular domain; NTC, Notch transcriptional activation complex; SHARP, silencing mediator of retinoid and thyroid hormone receptor/histone deacetylase 1-associated repressor protein; RBPJ, recombinant signal-binding protein for immunoglobulin κJ region; FHL1, four and a half LIM domain protein 1; MAML, mastermind-like; *HES*, hairy and enhancer of split; *HEY*, hairy and enhancer of split related with YRPW motif.

Notch signaling pathway is an ancient and highly conserved signaling cascade that is crucial for many biological processes (15). Aberrant Notch signaling pathway is associated with both noncancerous and cancerous diseases (16). The two core players in this signaling pathway are Notch receptors and their cognate ligands (17). In mammals, there are four Notch receptors (Notch1, Notch2, Notch3 and Notch4) and five Notch ligands (Dll1, Dll3, Dll4, Jag1 and Jag2) (18, 19). Notch receptors contain extracellular epidermal growth factor (EGF)-like domains mediating their association with Notch ligands (20). Notch ligands harbor a conserved Delta-Serrate ligand (DSL) domain executing ligand-receptor interaction (21). In signal-sending cells, Notch ligands are distributed on the cell membrane and interact with Notch receptors on signal-receiving cells (22) (**Figure 1**). In signal-receiving cells, Notch receptors are synthesized as precursor proteins by ribosomes bound to the endoplasmic reticulum (ER) and subsequently transferred to the Golgi apparatus (23). During this process, Notch receptors are glycosylated at the EGF-like repeat domain (24). In the Golgi apparatus, Notch receptors are cleaved by furin-like proteases at Site 1 (S1), generating a non-covalently associated heterodimer made up of an extracellular subunit and a transmembrane subunit (25). The N-terminal fragment of Notch receptors is O-glycosylated with the action of glycosyltransferase enzymes (e.g., members of the Fringe family) and transferred to the cell membrane (21). Notch receptors on the signal-receiving cell are activated by binding to Notch ligands on the cell surface of an adjacent signal-sending cell (26). Upon bound with ligands, Notch receptors are cleaved at Site 2 (S2) by a disintegrin and metalloprotease 10 (ADAM10) (27). The product of S2 cleavage is composed of a transmembrane domain and an intracellular domain (Notch extracellular truncation (NEXT)) (28). NEXT then undergoes proteolytic cleavage by γ-secretase at Site 3 (S3), liberating the Notch intracellular domain (NICD) that functions to regulate gene transcription (29). S3 processing can occur both on the plasma membrane and in the endosome after NEXT is endocytosed (30). After being released, NICD migrates into the cell nucleus where it forms a Notch transcriptional activation complex (NTC) with the DNA binding factor recombinant signal-binding protein for immunoglobulin κJ region (RBPJ) and transcriptional coactivators of the Mastermind-like (MAML) family (31). Following recruitment of transcriptional coregulators, NTC associates with Notch regulatory elements (NREs), fostering transcription of Notch target genes (4). The most conserved targets of Notch signaling include members of hairy and enhancer of split (HES) family and HES related with YRPW motif (HEY) family (32). In the absence of NICD, RBPJ combines with a variety of transcription repressors (e.g., four and a half LIM domain protein 1 (FHL1) and silencing mediator of retinoid and thyroid hormone receptor (SMRT)/histone deacetylase 1 (HDAC1)-associated repressor protein (SHARP)), enabling RBPJ to restrict the transcription of Notch target genes (33). The abundance of Notch ligands and receptors presented on cell membrane, the type of ligands, and glycosylation of the EGF domain affect ligand-receptor binding and the amount of released NICD (34).

Notch signaling is implicated in various aspects of cancer biology, such as the maintenance of cancer stem-like cells (CSCs), angiogenesis and tumor immunity (35, 36). Notch signaling acts as an oncogenic factor, while in some cancers its role may be tumor suppressive (37, 38). For instance, Dll1-mediated Notch signaling promoted the progression of breast cancer and glioma (39, 40). This signaling pathway exerted anticarcinogenic activities in lung cancer, osteosarcoma and pancreatic carcinoma (41–43). Notch signaling is necessary for CSC maintenance in diverse cancers, which can increase cancer heterogeneity, stemness, metastasis and resistance to anticancer therapies (35, 44). Notch signaling-mediated intercellular interactions contribute to induction of epithelial-mesenchymal transition (EMT) and remodeling of the tumor stroma (45, 46). Notch signaling has been implicated in vascular development and physiology (47). Accumulating evidence indicates that Notch ligand Dll4 is highly expressed in tumor vasculature and has a role in tumor angiogenesis (48, 49). Targeting Dll4 represents a prospective antiangiogenic strategy in cancer. Dll4 deficiency disturbed the dynamic balance of tumor angiogenesis and overcame resistance to vascular endothelial growth factor (VEGF) blockade in cancer cell line-based xenograft models (50, 51). Furthermore, Notch signaling has emerged as an important regulator of tumor immunity, resulting in tumor-promoting or tumor-antagonizing outcomes, which may depend on mobilization of Notch signaling in each form of tumor-infiltrating immune cells (52–54).

## 3 The interplay between Notch signaling and tumor immune microenvironment

**Figure 2:**
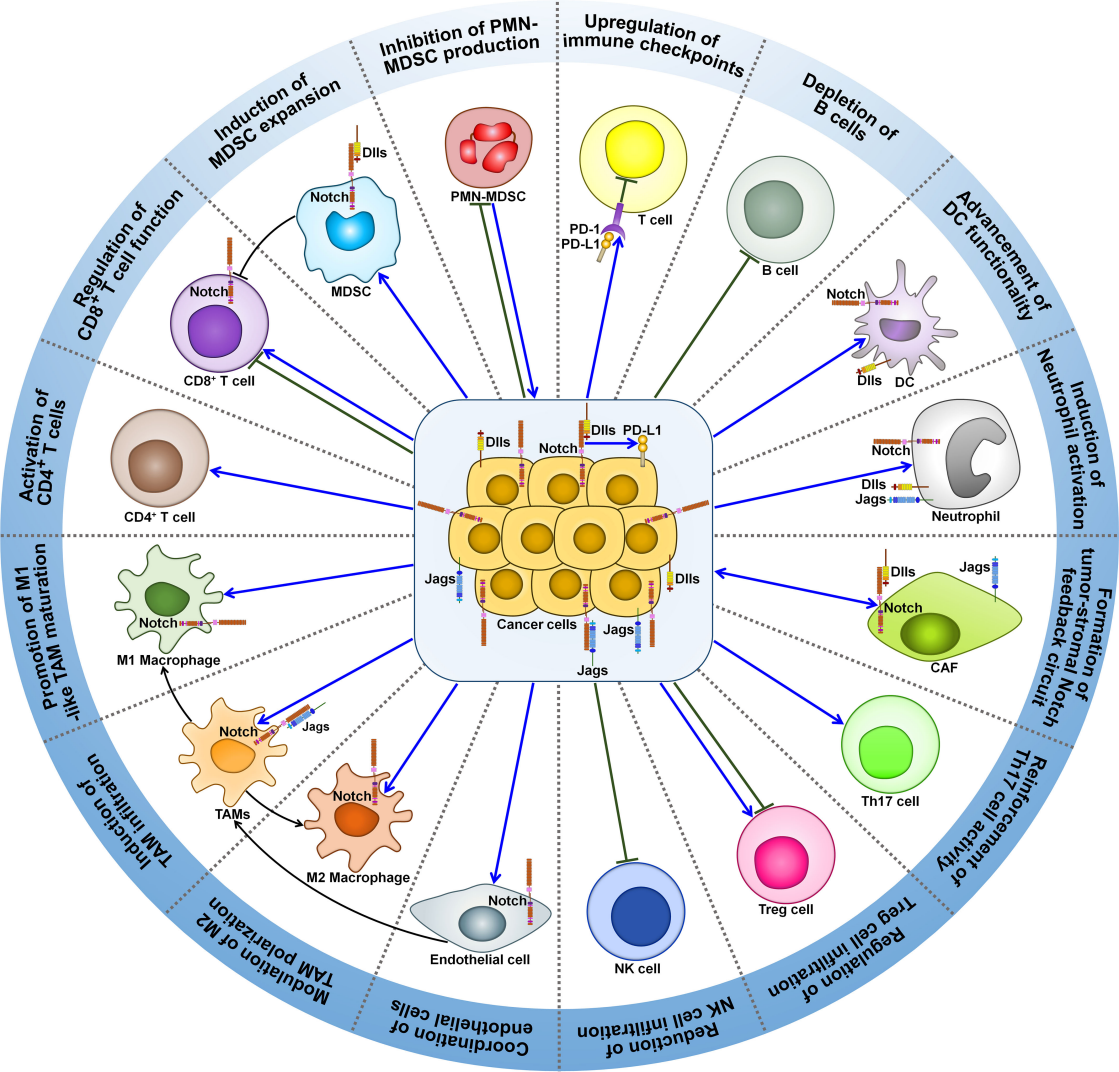
Roles of Notch signaling pathway in tumor immune microenvironment. Notch signaling can affect the activity of various immune cells within tumor microenvironment. Notch signaling suppresses PMN-MDSC production and induces MDSC expansion, contributing to tumor immunosuppression. Intriguingly, PMN-MDSCs can elicit Jag/Notch signal transduction pathway in cancer cell. The crosstalk between cancer cells and PMN-MDSCs finally enhances cancer metastasis. Notch signaling play dual roles in regulating T cell-mediated immune responses in cancer. Notch pathway can affect TAM recruitment and polarization. Cancer cells trigger the activation of Notch signaling in endothelial cells, which mediates the education of TAMs by cancer cells. This leads to formation of immunosuppressive microenvironment and impairment of T cell-mediated immunity. Notch activation reduces the intratumoral infiltration of NK cells, while it has opposite effects on Treg frequency and activity. Notch signaling leads to IL-17-driven inflammation via Th17 recruitment. Notch signaling mediates the communication between cancer cells and CAFs. On one hand, activated Notch pathway in CAFs causes compromised antitumor immunity and supports cancer development. On the other hand, increased activity of Notch pathway in CAFs can inhibit cancer progression. Cancer cells attract neutrophils into tumor microenvironment and motivate Notch signaling in recruited neutrophils. Notch-activated neutrophils suppress the cytotoxic activity of CD8^+^ T cells. Notch signaling fosters DC terminal differentiation and drives antigen cross-presentation to CD8^+^ T cells. Consequently, Notch-primed DCs retard cancer progression. Notch mutation is associated with a decline in B cell population in tumor microenvironment. Furthermore, Notch activation upregulates immune checkpoints (PD-1 and PD-L1) to blunt adaptive antitumor immunity. MDSC, myeloid-derived suppressor cell; Dlls, Delta-like ligands; PMN-MDSC, polymorphonuclear-myeloid-derived suppressor cell; PD-1, programmed cell death protein-1; PD-L1, programmed cell death protein-ligand 1; DC, dendritic cell; Jags, Jaggeds; CAF, cancer-associated fibroblast; Th17, T helper 17; Treg cell, regulatory T cell; NK cell, natural killer cell; TAMs, tumor-associated macrophages.

A growing body of evidence indicates that Notch signaling pathway can regulate the activity of various immune cells within tumor microenvironment (TME), such as MDSCs, TAMs, regulatory T cells (Tregs), cancer-associated fibroblasts (CAFs), DCs, neutrophils and T cells (**Figure 2**). Accordingly, Notch signaling is involved in cancer development through multifarious mechanisms, which include induction of cancer immunosuppression, promotion of tumor-supportive microenvironment, and regulation of adaptive antitumor immunity.

### 3.1 Regulation of immunosuppression

#### 3.1.1 Myeloid-derived suppressor cells

Notch1 signaling plays a critical role in tumor-induced immunosuppression. On one hand, Notch1 contributes to tumor immunosuppression through promotion of MDSCs. Notch-activated macrophages increased the liberation of C-C motif chemokine ligand 2 (CCL2) and interleukin-10 (IL-10) to induce MDSC recruitment in glioma (55). The MDSC-rich environment educated T cells to be immunosuppressive and attenuated the efficacy of oncolytic herpes simplex virus-1 (oHSV-1)-based virotherapy. Conversely, pharmacologic blockade of Notch reversed the immunosuppressive brakes and potentiated CD8^+^ T cell-driven antitumor immune responses, thereby improving oncolytic virus-induced therapeutic benefit. Deregulation of Notch signaling in immature T cells induced the expansion of CD11b^+^Gr-1^+^ MDSCs in T-cell acute lymphoblastic leukemia (T-ALL) through IL-6 (56). Depletion of MDSCs inhibited T-ALL cell proliferation and expansion. Notch3 increased the expression of various cytokines such as CCL2, colony-stimulating factor-1 (CSF-1) and C-X-C motif chemokine ligand 12 (CXCL12), which promoted recruitment of macrophages and MDSCs to the TME of colorectal cancer (CRC) (57). Therefore, Notch3 accelerated CRC development by favoring immunosuppressive TME. MDSCs were able to stimulate signal transducer and activator of transcription 3 (STAT3) and Notch in breast cancer cells through IL-6 and nitric oxide (NO), respectively (58). Notch activation supported sustained STAT3 activation via IL-6. The crosstalk between Notch and STAT3 signaling pathways mediated MDSC-induced breast cancer stemness. Notch signaling was strongly inhibited in MDSCs derived from colon carcinoma- and melanoma-bearing mice (59). Notch signaling suppressed the production of polymorphonuclear (PMN)-MDSCs but increased the generation of mononuclear MDSCs. Depletion of Notch signaling reduced the inhibitory effect of MDSCs on T cell function. Manipulation of Notch signaling cascade may represent a potential therapeutic option for cancer patients. PMN-MDSC-derived reactive oxygen species (ROS) activated Notch1 in circulating tumor cells (CTCs) isolated from patients with breast cancer or melanoma, which enabled CTCs to respond to Jag1-mediated Notch activation (60). The crosstalk between PMN-MDSCs and CTCs enhanced the dissemination and metastatic potentials of CTCs. On the other hand, Notch signaling counteracts MDSC-mediated immunosuppression. Activation of Notch/RBPJ signaling pathway blocked monocarboxylate transporter 2 (MCT2)-mediated lactate import in myeloid cells through its downstream molecule HES1 (61). This resulted in the suppression of MDSC differentiation and promotion of M1-like TAM maturation, thereby restricting lung cancer progression. MDSCs decreased the expression of Notch1 and Notch2 in T cells in a NO-dependent manner (62). Notch1 increased the cytotoxic effects of CD8^+^ T cells and induced their conversion into a memory phenotype. Restorement of Notch1 signaling in CD8^+^ T cells overcame MDSC-induced T cell suppression and improved the beneficial effects of T cell-based immunotherapy in lung cancer-bearing mice.

#### 3.1.2 Tumor-associated macrophages

TAMs play dual roles in remodeling of tumor immunosuppressive microenvironment. Notch induced the expression of IL-1β and CCL2, which then recruited TAMs to the tumor, contributing to breast cancer progression (63). High expression of Jag1/Notch1 were positively linked with infiltration levels of M2-like TAMs, contributing to poor prognosis in lung adenocarcinoma (LUAD) (64). Mechanistically, Notch signaling recruited macrophages into LUAD tissues through induction the secretion of CCL2 and IL-1β and promoted their polarization into M2 type. Jag1-mediated Notch signaling increased the secretion of IL-4 and IL-6 in breast cancer cells, leading to M2 polarization of TAMs and breast cancer development (65). Epithelial ovarian cancer (EOC) cells induced the activation of Notch pathway in endothelial cells to upregulate CD44 in TAMs, propelling their education by EOC cells (66). This resulted in formation of immunosuppressive microenvironment and impairment of T cell-mediated immunity, hence accelerating EOC progression. Notch signaling was activated in TAMs in the TME of pancreatic ductal adenocarcinoma (PDAC) (67). Notch activation favored TAM polarization into an immunosuppressive M2-like phenotype characterized by high levels of immunosuppressive cytokines (IL-10, transforming growth factor-β1 (TGF-β1) and tumor necrosis factor-α (TNF-α)), arginase 1 and immune checkpoint molecules. Notch inhibition cooperated with programmed cell death protein-1 (PD-1) blockade to induce CD8^+^ T cell-mediated antitumor immunity in PDAC. The combined treatment achieved a stronger anticancer effect than single treatment. Activation of Notch in triple negative breast cancer (TNBC) induced the expression of IL-1β and CCL2 to attract TAMs into the tumor, culminating in cancer development and immune evasion (68). Notch signaling in PDAC cells fostered the recruitment and activation of macrophages to a M2-like phenotype through upregulation of IL-8, CCL2, IL-1α and urokinase-type plasminogen activator (uPA) (69). In turn, activated TAMs then secreted IL-6 to enhance PDAC cell metastasis. Interruption of Notch-dependent circuit holds potential as a therapeutic option for PDAC treatment. Intriguingly, Notch signaling also limits the immunosuppressive function of TAMs. Myeloid-specific Notch activation remarkably reduced the infiltration of pro-tumorigenic M2 macrophages into PDAC tissues, which led to upregulated antigen presentation and enhanced cytotoxic T effector function (70). Notch signaling in myeloid cells suppressed the expansion of Kupffer cell-like TAMs (kclTAMs), thus inhibiting hepatocellular carcinoma (HCC) progression (71).

#### 3.1.3 Regulatory T cells

Notch1 overexpression favored melanoma growth *in vivo* (72). In terms of mechanism, Notch1 suppressed the intratumoral infiltration of CD8^+^ cytotoxic T lymphocytes (CTLs) and natural killer (NK) cells while increased the population of Tregs and MDSCs via upregulation of TGF-β1. It also inhibited T cell proliferation and activation and increased PD-1 expression on CD4^+^ and CD8^+^ T cells. High expression of Dll3 correlated with poor prognosis in patients with invasive breast cancer (73). Patients with high Dll3 expression had high proportions of T follicular helper (Tfh) cells and Tregs and low proportions of M2 macrophages and resting mast cells. The accumulation of tumor-promoting immune cells is likely to facilitate breast cancer pathogenesis. The infiltration of Tregs and T helper 17 (Th17) cells in the TME was increased with the development of gastric cancer, leading to an imbalance between Tregs and Th17 (74). CD4^+^CD25^+^CD127^dim/-^ Tregs expressing high levels of forkhead box protein P3 (FoxP3) function to maintain peripheral tolerance in cancer by inhibiting the activation of tumor-specific effector T cells (75). Retinoic acid-related orphan receptor γt (RORγt), a key transcription factor of IL-17, is expressed in Th17 cells and correlates with IL-17-driven inflammation (76). Notch signaling pathway was activated in gastric cancer (77). Inhibition of Notch signaling reduced the expression of transcription factors FoxP3/RORγt without affecting Treg/Th17 balance. Notch inhibition blunted the suppressive function of CD4^+^CD25^+^CD127^dim/-^ Tregs and IL-17 production by Th17 in gastric cancer. Altogether, Notch signaling played a critical role in regulating the activity of Tregs and Th17 cells during GC progression. Another study indicated that high Notch3 expression was associated with decreased infiltration of activated CD8^+^ T cells and emergence of immunosuppressive cells (e.g., Tregs and M2 macrophages) in the TME of gastric cancer (78). High Notch3 expression also contributed to upregulation of immune checkpoint genes (e.g., *CD200*, *CD274*, *CD276*, *CTLA4* and *HAVCR2*), leading to impaired immune responses. Altogether, Notch3 was involved in immune tolerance of gastric cancer, highlighting its potential as a therapeutic target for the treatment of gastric cancer.

### 3.2 Modification of tumor-supportive microenvironment

#### 3.2.1 Cancer-associated fibroblasts

Jag1/Notch2 signaling-mediated cell-to-cell contact between CAFs and lung cancer cells facilitated vascular mimicry (VM) generation and permeability (79). The loose junctions of VM networks fostered cancer intravasation and neutrophil penetration, leading to enhanced N2 neutrophil infiltration in lung cancer tissues. Blockade of Jag1/Notch2 interaction inhibited VM formation and lung cancer growth in a murine tumor model. Notch1 activation in TNBC cells promoted contact-dependent induction of CXCL8 in inflammation-induced TNBC-stromal interaction network, leading to TNBC metastasis and progression (80). CAF-derived stanniocalcin 1 (STC1) increased the stemness of HCC cells (81). Mechanistically, STC1 directly combined with Notch1 receptors to motivate the Notch signaling pathway. Moreover, Notch1 was capable of regulating STC1 at the transcriptional level, constituting a tumor-stromal amplifying STC1-Notch1 feedforward signal. Radiation induced the expansion of Dll1-overexpressing breast cancer CSCs, which impelled IL-6-dependent recruitment of CAFs within the TME (82). After that, Dll1-mediated Notch signaling in CAFs triggered Wnt signaling in Dll1-overexpressing CSCs to increase radioresistance in breast cancer. Moreover, blocking Dll1 and IL-6 sensitized Dll1-ovexpressing breast cancer cells to radiotherapy.

In contrast, Notch-activated CAFs can exert tumor-suppressive actions. Apoptotic lung cancer cells activated Notch1/Wnt-induced signaling protein-1 (WISP-1) signaling pathway in CAFs, thus inhibiting the migration and invasion of lung cancer cells (83). Intracellular Notch1 signaling in CAFs reduced the plasticity and stemness of melanoma stem/initiating cells (MICs), hence affecting cancer heterogeneity and aggressiveness (84). Increased activity of Notch pathway in melanoma-associated fibroblasts strikingly retarded cancer growth and angiogenesis (85). Activating Notch signaling in melanoma-associated fibroblasts may be a promising therapeutic strategy for melanoma treatment.

#### 3.2.2 Neutrophils

Previously, Wood et al. (86) reported that neutrophils with activated Notch and TGF-β signaling cascades were enriched in metastatic CRC tissues, suggesting the potential implication of neutrophils in formation of a pre-metastatic niche. Radiation exposure in lung tissues induced accumulation and activation of neutrophils, contributing to a series of tissue perturbations including persistent Notch activation in epithelial cells (87). Such changes facilitated a metastatic niche within irradiated lung tissues, accelerating the growth of arriving breast cancer cells. Notch ligands expressed by cancer cells or tumor-associated neutrophils (TANs) activated Notch1 receptors on endothelial cells (88). Endothelial Notch1 hyperactivation facilitated neutrophil infiltration and metastasis through upregulation of vascular cell adhesion molecule 1 (VCAM1), forming a pre-metastatic niche to drive cancer colonization. These events promoted circulating cancer cell migration, intravasation and homing at distant sites.

### 3.3 Coordination of adaptive antitumor immunity

#### 3.3.1 Dendritic cells

Jag2-induced Notch signaling could enhanced NK cell cytotoxicity mediated by DCs (89). This caused increased effector activity of cytotoxic T cells. Thus, overexpression of Jag2 retarded the proliferation of B cell lymphoma cells. Notch2 signaling was implicated in conventional DC (cDC)-mediated antitumor immunity (90). Depletion of Notch2 signaling dampened cDC terminal differentiation and inhibited antigen cross-presentation to CD8^+^ T cells through downregulation of chemokine receptor 7 (CCR7). Consequently, Notch-primed DCs inhibited the development of inflammation-associated colon cancer. Notch signaling in DCs prevents cancer progression by eliciting antitumor immune responses.

#### 3.3.2 Neutrophils

Unlike DCs, neutrophils function to impair T cell-mediated antitumor immune responses. Epithelial Notch1 induced TGF-β-dependent neutrophil recruitment into the TME of CRC, contributing to decreased infiltration of CD8^+^ T cells and cancer metastasis (91). EOC cells recruited TANs into the TME and triggered Jag2 expression in TANs via IL-8 release (92). Jag2-activated TANs dampened cytotoxic activity of CD8^+^ T cells. Blockade of Notch signaling inhibited EOC growth and strengthened the killing effect of CD8^+^ T cells.

#### 3.3.3 T cells

Notch signaling facilitates adaptive immune evasion in cancer. B cells, CD45^+^ cells, DCs, macrophages, mast cells, neutrophils and T cells were significantly reduced in Notch1-mutant relapsed T1-2N0 laryngeal cancer in comparison with wild-type cancer samples (93). Notch1-mutant recurrent tumors exhibited an immunosuppressive phenotype characterized by decreased B cells score, T cells score and tumor-infiltrating lymphocytes (TILs) score. Notch1 mutation leads to compromised immune response and immunosurveillance escape in recurrent laryngeal cancer. Tumor-derived Jag1 drove tumorigenesis through macrophage recruitment and functional repression of CD8^+^ T cells in breast cancer-bearing mice models (94). Jag1-induced Notch activation fostered the production of multiple cytokines including IL-6 and WISP-1, contributing to the recruitment of macrophages into the TME. Following recruitment, Notch-activated macrophages interacted with tumor-infiltrating T cells to suppress their proliferation and tumor-killing activity by enhancing CD14 and CD93 secretion. Combination of PD-1 blockade and a Notch inhibitor produced significantly greater inhibition of breast cancer growth than either individual agent. Jag1-mediated Notch activation propels adaptive immune evasion of cancer cells and offers novel therapeutic opportunities for cancer treatment. Inhibition of Notch activation reduced the production of proinflammatory cytokines (e.g., CCL2 and IL-1β) in TNBC (95). This triggered a switch to an immune-inflamed tumor phenotype characterized by a reduction in immunosuppressive Tregs and M2-polarized TAMs and increased infiltration of cytotoxic T cells into tumors. Notch signaling can program tumor immune landscape to facilitate TNBC immune evasion. Notch1 signaling decreased the expression of human leukocyte antigen (*HLA*) *class II* genes through downregulation of class II transactivator (*CIITA*) in chronic lymphocytic leukemia (CLL) cells (96). Notch1 also increased the expression of programmed cell death protein-ligand 1 (PD-L1) and promoted the exhaustion of CD8^+^ T cells. Notch1 signaling facilitated CLL escape from immune surveillance by blocking antigen presentation and inhibiting T cell activation.

Euchromatic histone methyltransferase 2 (EHMT2)-mediated activation of Notch1 signaling increased PD-L1 expression and reduced major histocompatibility complex-I (MHC-I) expression to dampen T cell immune responses, culminating in stemness maintenance of glioma stem cells (GSCs) (97). The binding of Jag1 expressed on tumor epithelial cells to Notch receptors on TAMs in PDAC microenvironment favored the activation of Notch signaling, which induced TAM polarization into an immunosuppressive phenotype (67). TAMs secreted immunosuppressive mediators (e.g., IL-10 and TGF-β) to suppress CD8^+^ T cell function. Furthermore, pharmacological inhibition of Notch signaling combined with PD-1 blockade led to reduced macrophage recruitment and increased CD8^+^ T cell activation, which led to tumor inhibition.

Notch signaling is capable of inducing T cell-mediated immune response (**Figure 2**). Reportedly, the transcriptional levels of Notch1/2/3 were increased in gastric cancer tissues compared with normal tissues (98). High transcription levels of Notch1/2/3/4 predicted a good prognosis in patients with gastric cancer. Upregulation of Notch family members was positively correlated with infiltration levels of CD4^+^ T cells, DCs, macrophages and neutrophils. Notch expression showed significant association with many immune markers in gastric cancer, including CCL2, CD163, C-X-C motif chemokine receptor 4 (CXCR4), CCR4 and IL-10. Notch1/2/3/4 might represent potential therapeutic targets for precision cancer treatment. Notch signaling pathway increased the expression of MHC-I and interferon-γ (IFN-γ)-dependent cytokines and favored the recruitment of antitumor CD8^+^ T cells in glioma (99). Moreover, Notch signaling promoted the conversion of TAMs into an anti-tumorigenic phenotype. Oppositely, blockade of Notch signaling facilitated tumor immune evasion and increased glioma cell aggressiveness. Collectively, Notch signaling pathway suppressed cancer development by remodeling tumor immune microenvironment. CRC patients with Notch mutation had higher infiltration levels of CD8^+^ T cells, M1 macrophages, neutrophils and NK cells (100). Notch signaling was positively associated with tumor immunogenicity in CRC. Notch mutant status predicted a good prognosis in CRC patients undergoing immune checkpoint inhibitor (ICI) treatment. Notch mutation is anticipated to act as a new therapeutic target and prognostic factor for CRC. Another study indicated that mutated Notch signaling correlated with an enrichment of tumor-specific CD8^+^ T cells and deprivation of Tregs (101). Therefore, Notch signaling pathway mutations augmented antitumor immunity in CRC. The expression levels of *Notch1*, *Notch3* and *Notch4* were remarkably decreased in prostate cancer tissues compared with normal tissues (102). The abundance of *Notch* family genes was connected with intratumoral infiltration of CD4^+^ T cells and CD8^+^ T cells, which influenced clinical outcomes in patients with prostate cancer. Silencing of Notch ligand Dll1 on DCs dampened antitumor immune responses in lung and pancreatic cancers by inhibiting effector CD8^+^ T cell function and T effector memory (Tem) cell differentiation as well as promoting the accumulation of Tregs and MDSCs (103). Consequently, pharmacological inhibition of Dll1 promoted the growth of lung and pancreatic cancers. In addition, enforced expression of Jag1 reduced Treg frequency and enhanced antitumor immunity through downregulation of PD-1 on CD8^+^ Tem cells. Notch ligands are crucial for triggering antitumor T cell immune responses to prevent cancer development.

#### 3.3.4 Immune checkpoint molecules

Immune checkpoints refer to as a class of stimulatory and inhibitory molecules expressed on immune cells and cancer cells (104). They act as accessory molecules that either induce or suppress T cell activation (105). PD-1 is an inhibitory receptor expressed on T cells, while PD-L1 is a ligand of PD-1 (106). The PD-L1/PD-1 signaling cascade constitutes an adaptive immune resistance mechanism in cancer. Notch signaling was responsible for PD-L1 upregulation, contributing to immune evasion in gastric cancer (107). Notch/c-Myc/enhancer of zeste homolog 2 (EZH2) signaling induced PD-L1 expression in CLL, contributing to enhanced resistance to activated autologous T cells in a murine CLL model (108). Likewise, Notch3 was upregulated in PD-L1-overexpressing breast CSCs (109). Notch3 activation increased PD-L1 expression by regulating the activity of mammalian target of rapamycin (mTOR), which was crucial for maintaining the stemness of CSC-like cells. High expression levels of Dll3 were inversely linked with Notch1 but showed significant association with high expression of PD-L1 (110). This resulted in dysfunction of T lymphocytes. However, high Dll3 expression was associated with increased infiltration of immune cells and exerted a protective effect on both progression-free survival (PFS) and overall survival (OS) in PDAC patients.

## 4 Effects of Notch signaling on cancer immunotherapy response

Due to its role in tumor immunity, Notch signaling affects immunotherapy response in cancer. Reduced Notch activity in glioma impaired MHC-I expression and inhibited recruitment of IFN-γ-producing immune cells, contributing to tumor immune evasion (99). The TME of Notch-inhibited glioma was characterized by an exhaustion of T cells and homeostatic microglia-like TAMs and an enrichment of immunosuppressive TAMs. Loss of Notch activity accounted for increased resistance to CSF-1 receptor (CSF-1R) inhibition in glioma. A risk signature consisting of six Notch pathway-related genes (*CNTN1*, *DTX3L*, *ENO1*, *GATA3*, *MAGEA1* and *SORBS2*) acted as an independent prognostic factor in bladder cancer (111). The infiltrating levels of immune cells were markedly distinct among different risk groups. The high-risk group had higher levels of macrophages and resting mast cells, while CD8^+^ T cells, DCs, monocytes and Tfh cells were enriched in low-risk patients. Furthermore, low-risk patients displayed a better response for immunotherapy. Thus, Notch signaling was linked with the immune status and immunotherapy response in bladder cancer.

Notch4 mutation was associated with enhanced immunogenicity and increased infiltration of TILs and CD8^+^ T cells in patients with diverse types of cancer (112). Patients with Notch4 mutation had a better response to ICI therapy. High-mutated Notch signaling was positively associated with increased levels of M1 macrophages, activated memory CD4^+^ T cells and CD8^+^ T cells in non-small cell lung cancer (NSCLC) (113). It also negatively correlated with M2 macrophages, quiescent mast cells and resting memory CD4^+^ T cells. High-mutated Notch signaling was linked with inflammatory immune microenvironment and increased immunogenicity. As expected, patients with high-mutated Notch signaling benefited more from ICI therapy than those with low quantities of mutations in Notch signaling. High-mutated Notch signaling could serve as a predictive biomarker of the response to immunotherapy in NSCLC patients. Co-occurring mutations in Notch and homologous repair (HR) pathways correlated with enhanced efficacy of ICIs and longer PFS in patients with advanced NSCLC (114). Co-occurring alteration of DNA damage response (DDR) and Notch pathways was associated with increased infiltration of CD4^+^ T cells in NSCLC patients (115). Mutated DDR and Notch pathways predicted superior survival outcomes in ICI-treated NSCLC patients.

Loss-of-function mutation of Notch signaling increased the expression of chemokines (e.g., CXCL1 and C-X3-C motif chemokine ligand 1 (CX3CL1)) in CRC, which then recruited both pro-tumor (e.g., MDSCs and neutrophils) and antitumor immune cells (B cells, CD4^+^ T cells, CD8^+^ T cells, DCs and NK cells) (116). Notch mutation correlated with good prognosis for CRC patients undergoing ICI therapy. Blocking Notch signaling can reinforce antitumor immunity by affecting the chemotaxis of various immune cells. Activation of TGF-β and Notch pathways was linked with response to ICI and poor therapeutic outcomes in CRC patients (117). Pharmacological blockade of TGF-β and Notch pathways overcame resistance to PD-1 inhibitor in CRC.

## 5 The anticancer activity of Notch-targeted treatments

As a classical signaling cascade in humans, Notch signaling is indispensable for the homeostasis and development of most tissues. Dysregulation of Notch signaling contributes to carcinogenesis and cancer pathogenesis. Many notch-directing therapies have been developed and tested in preclinical and clinical studies, including antibodies targeting Notch ligands or receptors, small molecule inhibitors of Notch receptors and GSI (**Table 1**).

**Table 1:** List of Notch-targeted therapies in preclinical and clinical studies.

Type of drug	Drug	Type of study	Cancer type	Number of subjects	Results	Reference
Antibodies against Notch ligands	Anti-Jag1 antibody	Preclinical study	Triple negative breast cancer	–	Suppress cancer stem cells and delay cancer growth	(118)
	Dl1.72	Preclinical study	Breast cancer	–	Inhibit the proliferation and liver metastasis of cancer cells	(119)
	Demcizumab	Phase 1b open-label study	Epithelial ovarian cancer	19	Have a manageable toxicity profile; result in clinical benefit rate of 42%	(120)
Antibodies against Notch receptors	EGFR/Notch bispecific antibodies	Preclinical study	Non-small cell lung cancer	–	Reduce the proportion of stem-like cells; decrease the frequency of tumor-initiating cells; overcome chemotherapy resistance	(121)
	Notch 1 antibody	Preclinical study	Triple negative breast cancer	–	Reduce tumor burden; extend animal survival	(122)
	Tarextumab	Phase 1 dose escalation and expansion study	Advanced solid tumors	42	Show good tolerability; lead to disease stabilization in nine subjects	(123)
	Tarextumab	Phase 2, randomized, placebo-controlled, multicenter trial	Pancreatic cancer	177	Show limited clinical activity	(124)
Notch inhibitor	ASR490	Preclinical study	Breast cancer	–	Restrain cancer growth	(125)
	G9	Preclinical study	Triple negative breast cancer	–	Enhance antitumor immune responses; restrain cancer growth; have a favorable safety profile	(95)
	NADI-351	Preclinical study	Esophageal adenocarcinoma	–	Restrain cancer growth	(126)
	DAPT	Preclinical study	Pancreatic ductal adenocarcinoma	–	Restrain cancer growth	(127)
	DAPT	Preclinical study	Osteosarcoma	–	Restrict cancer metastasis	(128)
	CB-103	Preclinical study	T-cell acute lymphoblastic leukemia	–	Extend overall survival	(129)
	CB-103	Open-label, nonrandomized, phase 1/2 dose-escalation study	Adenoid cystic carcinoma	79	Have a manageable safety profile; show limited clinical anticancer activity	(130)
Antibody-drug conjugates	Notch3-ADCs	Preclinical study	Ovarian cancer	–	Outperform stand-of-care chemotherapy; lead to sustained tumor regressions	(131)
	Rova-T	Preclinical study	*IDH*-mutant glioma	–	Exhibit cytotoxic activity against glioma tumorspheres	(132)
	Rova-T	Open-label, two-to-one randomized, phase 3 study	Small cell lung cancer	444	Show limited clinical activity	(133)
γ-secretase inhibitor	Dibenzazepine	Preclinical study	Mucoepidermoid carcinoma	–	Restrain cancer growth	(134)
	Dibenzazepine	Preclinical study	Bladder cancer	–	Restrain cancer growth	(135)
	RO4929097	Phase II, open label, nonrandomized trial	Glioblastoma	47	Exhibit minimal inhibition of neurosphere formation in cancer tissues	(136)
	RO4929097	Phase 1b dose escalation trial	ERα-positive metastatic breast cancer	15	Result in a total clinical benefit rate of 20% and a progression-free survival of 3.2 months	(137)
	RO4929097	Phase 1b/2 randomized study	Advanced sarcoma	76	Result in a progression-free survival of 8.9 weeks and an overall survival of 11.9 months	(138)
	RO4929097	Phase 1 study	Triple negative breast cancer	14	Show an overall response rate of 64% and pathologic complete response rate of 36%	(139)
	Crenigacestat	Phase 1 multicenter, nonrandomized, open-label trial	Adenoid cystic carcinoma	22	Elicit manageable toxicity; show limited clinical anticancer activity	(140)
	Crenigacestat	Phase 1, single-center, nonrandomized, single-arm, open-label, dose-escalation study	Advanced solid tumors	11	Have good tolerability; show limited clinical anticancer activity	(141)
	Crenigacestat	Multicenter, nonrandomized, open-label, phase 1b study	Advanced or metastatic solid tumors	63	Show limited clinical anticancer activity	(142)
	Crenigacestat	Multicenter, nonrandomized, open-label, phase 1b study	Advanced or metastatic solid tumors	31	Show limited clinical anticancer activity	(143)
	Crenigacestat	Multicenter, nonrandomized, open-label, dose-escalation, phase 1 study	T-cell acute lymphoblastic leukemia/T-cell lymphoblastic lymphoma	36	Show limited clinical anticancer activity	(144)
	Crenigacestat	Multicenter, nonrandomized, open-label, phase 1 study	Advanced or metastatic solid tumors/lymphoma	28	Induce disease stabilization in 54.5% and 64.7% of patients	(145)

### 5.1 Antibodies against Notch ligands

Jag1-targeting antibody blocked Notch signaling, suppressed TNBC CSCs and delayed tumor growth in preclinical brain metastasis xenograft models (118). Moreover, Jag1-neutralizing antibody treatment did not give rise to any detectable toxicity, suggesting its potential clinical application in cancer treatment. Jag1-based immunotherapy may represent a future treatment strategy in metastatic breast cancer. Anti-Dll1 antibody (Dl1.72) showed high affinity and specificity for Dll1 and markedly blocked Dll1/Notch signaling cascade (119). Dl1.72 inhibited the proliferation and liver metastasis of breast cancer cells in a xenograft mouse model. Dl1.72 did not cause detectable toxicity. An open-label phase 1b clinical trial was conducted in patients with platinum-resistant EOC (120). Demcizumab (Dll4-targeted IgG2 humanized monoclonal antibody) combined with paclitaxel possessed a manageable toxicity profile and resulted in clinical benefit rate of 42% in the 19 treated patients.

### 5.2 Antibodies against Notch receptors

Epidermal growth factor receptor (EGFR)/Notch-targeting bispecific antibodies reduced the proportion of stem-like cells, decreased the frequency of tumor-initiating cells, and overcame resistance to cetuximab and talazoparib in a NSCLC patient-derived xenograft (PDX) model (121). Moreover, combinatorial treatment of EGFR/Notch-targeting bispecific antibodies and talazoparib produced a stronger antitumor effect than the combination of cetuximab and talazoparib in epithelial tumors. Nanoparticle-mediated co-delivery of Notch1-targeting antibodies and small molecule drug ABT-737 decreased tumor burden and extended animal survival in a murine model of TNBC xenograft tumors (122). The therapeutic potential of this treatment merits thorough investigation. Tarextumab (OMP-5948), a new cross-reactive antibody that selectively targets Notch2 and Notch3, was well-tolerated in 42 enrolled patients with advanced solid tumors (123). Disease stabilization was observed in nine tarextumab-treated subjects. A randomized phase II trial demonstrated that addition of tarextumab to gemcitabine and nab-paclitaxel did not improve clinical outcomes over conventional chemotherapy in patients with untreated metastatic PDAC (124). The efficacy of tarextumab needs to be tested in further clinical studies.

### 5.3 Notch inhibitor

The Notch1 inhibitor ASR490 was reported to suppress the growth of breast cancer stem cells (BCSCs) and breast cancer in xenograft models (125). ASR490 may represent an attractive therapeutic agent against breast cancer, necessitating further clinical validation. Pharmacological inhibition of Notch signaling using G9 augmented antitumor immune responses and suppressed cancer growth with a favorable safety profile in a murine TNBC model (95). Notch1-selective small molecule inhibitor NADI-351 significantly restricted the growth of esophageal adenocarcinoma (EAC) with limited toxicity in PDX models through ablation of the CSC population (126). NADI-351 may be a promising therapeutic agent for cancer treatment. Both IL-17 signaling and Notch pathway accelerated PDAC progression, and IL-17 increased the activity of Notch signaling via nuclear factor-κB (NF-κB) pathway (127). Knockout of IL-17 and Notch inhibitor DAPT (N-[N-(3,5-difluorophenacetyl)-L-alanyl]-S-phenylglycine t-butyl ester) exerted synergistic effects against PDAC *in vivo*. The anticarcinogenic mechanism of IL-17-Notch co-inhibition needs to be adequately deciphered. Sublethal concentrations of cisplatin promoted osteosarcoma cell migration and invasion by activating the Notch signaling (128). Combination treatment of cisplatin with Notch inhibitor DAPT restrained osteosarcoma cell metastasis in xenograft tumor models.

CB-103 is an orally active small molecule that acts to impede Notch signaling (146). CB-103 combined with protein kinase B (Akt) inhibitor (MK-2206) strikingly extended OS in a xenograft model of T-ALL compared with the single agent (129). It is worthwhile to consider the further development of this combination treatment as a therapeutic option to improve clinical outcomes in T-ALL patients. In an open-label, nonrandomized, phase 1/2 dose-escalation study, CB-103 exhibited limited therapeutic benefits as monotherapy in patients with adenoid cystic carcinoma (ACC) (130). Future studies exploring the anticancer activity of CB-103 combined with current cancer treatments are required.

### 5.4 Antibody-drug conjugates

Notch3-targeted antibody-drug conjugates (Notch3-ADCs) outperformed conventional chemotherapy (carboplatin) and resulted in persistent cancer regressions in ovarian cancer-bearing mice (131). Continued study will be necessary to evaluate the clinical efficacy of this Notch-targeting treatment. An antibody-drug conjugate (Rova-T) comprising an anti-Dll3 antibody tethered to a chemotherapeutic drug pyrrolobenzodiazepine showed cytotoxic effects on Dll3-expressing patient-derived isocitrate dehydrogenase (*IDH*)-mutant glioma (132). An open-label, two-to-one randomized, phase 3 study was previously conducted to explore the anticancer benefit of Rova-T in patients with small cell lung cancer (SCLC) (133). However, patients receiving Rova-T had a shorter OS than the second-line chemotherapeutic drug topotecan.

### 5.5 γ-secretase inhibitor

The GSI dibenzazepine (DBZ)-mediated suppression of Notch signaling inhibited the growth of mucoepidermoid carcinoma (MEC) *in vivo* (134). Furthermore, co-targeting Notch and EGFR signalings exhibited an enhanced inhibitory effect on MEC growth. High Notch activity promoted bladder cancer development (135). The GSI DBZ and the mitogen-activated extracellular signal-regulated kinase (MEK) inhibitor selumetinib (AZD) were used to block Notch and MAPK signaling pathways, respectively. Combined blockade of these signaling pathways significantly repressed tumor growth in a murine xenograft model of bladder cancer. RO4929097, an oral GSI, blocked the Notch signaling pathway (136). It exerted limited inhibitory effects on the formation of brain tumor neurospheres in glioblastoma (GBM) patients in a phase 2 and pharmacodynamic trial. A phase 1b dose escalation trial of RO4929097 with exemestane indicated that this combination treatment led to a total clinical benefit rate of 20% and a PFS of 3.2 months in 14 patients with estrogen receptor α (ERα)-positive metastatic breast cancer (137). Combination of GSI and endocrine treatment warrants additional investigation in endocrine-resistant ERα-positive breast cancer. RO4929097 combined with vismodegib had good tolerability in 33 patients with advanced sarcoma (138). RO4929097 monotherapy led to a PFS of 8.9 weeks and an OS of 11.9 months. However, the administration of vismodegib did not reinforce the clinical activity of RO4929097. RO4929097 in conjunction with carboplatin and paclitaxel exhibited a good safety profile in patients with stage II-III TNBC (139). This combinatorial regimen demonstrated an overall response rate of 64% and pathologic complete response rate of 36% in TNBC patients.

The safety and anticancer effect of crenigacestat (LY3039478), a GSI as a selective oral Notch inhibitor, were evaluated in phase I trials (140, 141). Crenigacestat elicited limited clinical activity with a manageable safety profile in patients with advanced solid tumors. Likewise, a phase 1b study showed that crenigacestat combined with anticancer agents (abemaciclib, LY3023414, taladegib, carboplatin, cisplatin or gemcitabine) resulted in disappointing clinical efficacy in patients with advanced or metastatic solid tumors (142, 143). A multicenter, nonrandomized, open-label, dose-escalation, phase 1 trial revealed that crenigacestat showed low clinical efficacy in patients with relapsed/refractory T-ALL and T-cell lymphoblastic lymphoma (T-LBL) (144). The combination of crenigacestat and prednisone induced disease stabilization in 54.5% and 64.7% of patients with advanced or metastatic solid tumors or lymphoma that received different dosing schedules (145).

Taken together, the overall performance of Notch-directed therapies in clinical studies does not meet expectations. The clinical translation of Notch-targeted therapies has faced unneglectable challenges. A primary obstacle to their clinical use is likely to be their adverse effects. The use of Notch-targeting agents as a combination therapy or other strategies that manipulate dosing regimens would be helpful to alleviate or avoid their toxicity. The role of Notch signaling varies depending on the type of cancer and its crosstalk with other signaling cascades. Therefore, an in-depth investigation of Notch signaling in cancer biology will open the door to new options in cancer treatment.

## 6 Conclusions and future perspectives

Notch signaling modulates various aspects of tumor immunity including myeloid compartment functionality and T cell activation. Increasing knowledge of Notch signaling in immune cell regulation and function can shed new lights on future directions for clinical management of malignant tumors that are recalcitrant to standard therapies. Currently, the complicated roles of Notch signaling in tumor immune responses remain largely understudied. It is acknowledged that Notch signaling can regulate immune cells involved in pro-tumor or antitumor responses. Thus, Notch signaling can be tumor-promoting or tumor-suppressive, yet the inducing factors or conditions resulting in certain effects are still unclear. Relative abundance of immune cells, cytokines, chemokines, magnitude of Notch activation, and the type of Notch-activated cells may affect Notch-mediated immune regulation, which warrants future research attention. Notch signaling is generally activated among cells to mediate intercellular crosstalk, but its effects extremely rely on cancer type. The overall effect of Notch signaling on tumor immune microenvironment must be carefully detected in diverse types of cancer. In addition, Notch mutation is linked with immune cell infiltration within TME (101). It is equally important to define distinct Notch mutations and their influence on tumor immune microenvironment.

So far, some Notch-targeting drugs (e.g., Notch inhibitors, ligand/receptor-targeting antibodies or antibody-drug conjugates) have been evaluated in clinical trials. Nevertheless, there are still many obstacles, hindering the successful implementation of Notch-targeted therapeutics, such as insufficient anticancer efficacy, off-target toxicity and resistance to therapy. Pan-Notch inhibitors and limited affinity of antibody-drug conjugates might be plausible explanations for the off-target toxicity. Attenuating drug toxicities, using intermittent dosing regimens and developing multi-targeted agents are expected to be useful strategies to address this issue. Non-specific Notch inhibitors might simultaneously block Notch signaling pathway in cancer cells and immune cells, thus resulting in opposite effects on cancer control. To improve tumor targetability and agent efficacy, it is imperative to exploit new strategies for efficient delivery of Notch-targeting drugs to cancer cells. Nanocarrier systems such as dendrimers, liposomes or nanoparticles can be exploited to encapsulate, protect and target Notch-targeting drugs to the tumor site. Moreover, the perplexity of Notch signaling and bypass signaling pathways may restrict the clinical activity of Notch-targeted drugs, which highlights the need for further research on Notch regulatory networks in cancer. Notch mutation and activation status are associated with cancer pathogenesis and resistance to therapy. However, the Notch mutation and/or activation status in enrolled subjects were usually elusive in previous clinical trials. Therefore, it is necessary to monitor Notch activation status and levels in cancer patients in further clinical trials. Identifying predictive biomarkers for patient stratification is essential for the clinical development of Notch-targeting drugs. There is an urgent need to discover more predictive biomarkers for response to Notch-based therapies in cancer patients. Notch-targeted therapies can enhance response to conventional anticancer treatments (e.g., chemotherapy and immunotherapy). Notch inhibitors in conjunction with other treatment modalities can lead to synergistic anticancer effects. The combination treatment strategies hold great promise for therapeutic applications in cancer.

In conclusion, Notch signaling has emerged as a key regulator in tumor immunity. It can control the activity of various cells within TME. Therefore, targeting Notch signaling appears an effective approach to advance the clinical benefits of immunotherapy. However, much work remains to thoroughly understand Notch signaling and its effects on tumor immune microenvironment. Comprehensive insights into molecular mechanisms of Notch signaling in oncoimmunology may facilitate the development of more innovative and precise targeted therapies that will improve clinical outcomes in cancer patients.

## Author contributions

MW: Conceptualization, Supervision, Visualization, Writing – original draft. FY: Investigation, Resources, Writing – review & editing. YZ: Resources, Writing – review & editing. PL: Conceptualization, Supervision, Writing – review & editing.

## Glossary

**Table:**

CSCs	cancer stem-like cells
TME	tumor microenvironment
Dll1	Delta-like ligand 1
Dll3	Delta-like ligand 3
Dll4	Delta-like ligand 4
Jag1	Jagged1
Jag2	Jagged2
MDSCs	myeloid-derived suppressor cells
TAMs	tumor-associated macrophages
DCs	dendritic cells
GSI	γ-secretase inhibitor
EGF	epidermal growth factor
DSL	Delta-Serrate ligand
ER	endoplasmic reticulum
S1	Site 1
S2	Site 2
ADAM10	a disintegrin and metalloprotease 10
NEXT	Notch extracellular truncation
S3	Site 3
NICD	Notch intracellular domain
NTC	Notch transcriptional activation complex
RBPJ	recombinant signal-binding protein for immunoglobulin κJ region
MAML	Mastermind-like
NREs	Notch regulatory elements
HES	hairy and enhancer of split
HEY	hairy and enhancer of split related with YRPW motif
FHL1	four and a half LIM domain protein 1
SMRT	silencing mediator of retinoid and thyroid hormone receptor
HDAC1	histone deacetylase 1
SHARP	silencing mediator of retinoid and thyroid hormone receptor/histone deacetylase 1-associated repressor protein
EMT	epithelial-mesenchymal transition
VEGF	vascular endothelial growth factor
Tregs	regulatory T cells
CAFs	cancer-associated fibroblasts
CCL2	C-C motif chemokine ligand 2
IL-10	interleukin-10
oHSV-1	oncolytic herpes simplex virus-1
T-ALL	T-cell acute lymphoblastic leukemia
CSF-1	colony-stimulating factor-1
CXCL12	C-X-C motif chemokine ligand 12
CRC	colorectal cancer
STAT3	signal transducer and activator of transcription 3
NO	nitric oxide
PMN	polymorphonuclear
ROS	reactive oxygen species
CTCs	circulating tumor cells
MCT2	monocarboxylate transporter 2
LUAD	lung adenocarcinoma
EOC	epithelial ovarian cancer
PDAC	pancreatic ductal adenocarcinoma
TGF-β1	transforming growth factor-β1
TNF-α	tumor necrosis factor-α
PD-1	programmed cell death protein-1
TNBC	triple negative breast cancer
uPA	urokinase-type plasminogen activator
kclTAMs	Kupffer cell-like tumor-associated macrophages
HCC	hepatocellular carcinoma
CTLs	cytotoxic T lymphocytes
NK	natural killer
Tfh	T follicular helper
Th17	T helper 17
FoxP3	forkhead box protein P3
RORγt	retinoic acid-related orphan receptor γt
VM	vascular mimicry
STC1	stanniocalcin 1
WISP-1	Wnt-induced signaling protein-1
MICs	melanoma initiating cells
TANs	tumor-associated neutrophils
VCAM1	vascular cell adhesion molecule 1
cDC	conventional dendritic cell
CCR7	chemokine receptor 7
TILs	tumor-infiltrating lymphocytes
*HLA*	human leukocyte antigen
*CIITA*	class II transactivator
CLL	chronic lymphocytic leukemia
PD-L1	programmed cell death protein-ligand 1
EHMT2	euchromatic histone methyltransferase 2
MHC-I	major histocompatibility complex-I
GSCs	glioma stem cells
CXCR4	C-X-C motif chemokine receptor 4
IFN-γ	interferon-γ
ICI	immune checkpoint inhibitor
Tem	T effector memory
EZH2	enhancer of zeste homolog 2
mTOR	mammalian target of rapamycin
PFS	progression-free survival
OS	overall survival
CSF-1R	colony-stimulating factor-1 receptor
NSCLC	non-small cell lung cancer
HR	homologous repair
DDR	DNA damage response
CX3CL1	C-X3-C motif chemokine ligand 1
EGFR	epidermal growth factor receptor
PDX	patient-derived xenograft
BCSCs	breast cancer stem cells
EAC	esophageal adenocarcinoma
NF-κB	nuclear factor-κB
DAPT	N-[N-(3,5-difluorophenacetyl)-L-alanyl]-S-phenylglycine t-butyl ester
Akt	protein kinase B
ACC	adenoid cystic carcinoma
ADCs	antibody-drug conjugates
*IDH*	isocitrate dehydrogenase
SCLC	small cell lung cancer
DBZ	dibenzazepine
MEC	mucoepidermoid carcinoma
MEK	mitogen-activated extracellular signal-regulated kinase
AZD	selumetinib
GBM	glioblastoma
ERα	estrogen receptor α
T-LBL	T-cell lymphoblastic lymphoma
